# Ischemic Stroke of Suspected Cardioembolic Origin Despite Anticoagulation: Does Thrombus Analysis Help to Clarify Etiology?

**DOI:** 10.3389/fneur.2022.824792

**Published:** 2022-03-11

**Authors:** Benno Ikenberg, Tobias Boeckh-Behrens, Christian Maegerlein, Johanna Härtl, Moritz Hernandez Petzsche, Claus Zimmer, Silke Wunderlich, Maria Berndt

**Affiliations:** ^1^Department of Neurology, School of Medicine, Klinikum rechts der Isar, Technical University of Munich, Munich, Germany; ^2^Department of Diagnostic and Interventional Neuroradiology, School of Medicine, Klinikum rechts der Isar, Technical University of Munich, Munich, Germany

**Keywords:** thrombus, stroke, anticoagulation, histology, perviousness, cardioembolism, thrombectomy

## Abstract

**Introduction:**

Despite sufficient oral anticoagulation (OAC) to prevent cardioembolism, some patients suffer from cerebral ischemic strokes of suspected cardioembolic origin. Reasons for that are not clarified yet. In certain cases, the suspected cardioembolic origin of stroke is questioned. This study aimed to understand the thrombi origin and pathophysiology in patients suffering from stroke despite OAC by the analysis of histologic thrombus composition and imaging characteristics.

**Materials and Methods:**

On two distinct cohorts, we retrospectively analyzed histologic (*n* = 92) and imaging features (*n* = 64), i.e., thrombus perviousness in admission CT imaging, of cerebral thrombi retrieved by the endovascular treatment for a large vessel occlusion of the anterior circulation. In each group, patients with non-cardioembolic strokes and suspected cardioembolic strokes with or without anticoagulation were compared.

**Results:**

Fibrin/platelet content of suspected cardioembolic thrombi (mean/SD 57.2% ± 13) is higher than in non-cardioembolic thrombi (48.9% ± 17; *p* = 0.01). In suspected cardioembolic thrombi, the fibrin/platelet content does not differ in the subgroups of patients with (57.3% ± 13) and without prior OAC treatment (56.6% ±13; *p* = 0.8), both with higher values than non-cardioembolic thrombi. Thrombus perviousness (ε) of suspected cardioembolic OAC thrombi (mean/SD: 0.09 ± 0.06) differs significantly from non-cardioembolic thrombi (0.02 ± 0.02; *p* < 0.001). Further, ε is higher in suspected cardioembolic thrombi with OAC than in cardioembolic thrombi without OAC (0.06 ± 0.03; *p* = 0.04) and with insufficient OAC (0.04 ± 0.02; *p* = 0.07).

**Conclusion:**

Thrombi of the suspected cardioembolic origin of patients with prior OAC do not differ in their histologic composition from those without prior OAC, but both differ from non-cardioembolic thrombi. These histologic results make a non-cardioembolic etiology for strokes despite prior OAC rather unlikely but favor other reasons for these ischemic events. Perviousness assessment reinforces the histologic findings, with additional information about the OAC thrombi, which present with higher perviousness. This suggests that OAC would not affect the relative histologic thrombus composition but may alter the microstructure, as reflected by perviousness.

## Introduction

Cerebral ischemic stroke due to a large vessel occlusion is often caused by cardioembolism. For prophylaxis of patients with cardioembolic stroke, mostly receive oral anticoagulation (OAC) (1–3). However, despite sufficient medication, some patients suffer from cerebral ischemic strokes of the suspected cardioembolic origin (4). These patients are at higher risk for further stroke recurrence (4, 5), which illustrates the importance of a better pathophysiologic understanding of these ischemic events despite therapy, as reasons for that are not clarified yet. Phenomena, such as non-adherence to medication, genetic predisposition regarding drug effectiveness, or lastly another underlying stroke cause than the suspected cardioembolic origin have been discussed (4, 6, 7). So far, selection of an appropriate secondary prophylaxis to prevent the stroke recurrence in such patients remains difficult for the attending stroke physicians.

Etiologic stroke work-up includes, among others, Holter-monitoring, laboratory analysis, ultrasound of the brain supplying blood vessels, and echocardiography (2). In addition to this routine work-up, the histologic analysis of cerebral thrombi is now widely available and may be helpful to determine the underlying stroke cause (8, 9). Furthermore, an imaging analysis of thrombus characteristics (such as, thrombus perviousness measurement) may be helpful to determine the underlying stroke cause or optimize recanalization procedure (10–12).

With regard to an increased risk of stroke recurrence after an ischemic event despite anticoagulation for a suspected cardioembolic source (4, 5), the important question remains which is the appropriate secondary prophylaxis for those patients. In the present study, the histologic and imaging characteristics of thrombi from patients with an acute ischemic stroke despite anticoagulation for treatment of a suspected cardioembolic source were analyzed and compared with suspected cardioembolic strokes without anticoagulation as well as patients with non-cardioembolic stroke. This study aimed to evaluate the underlying pathophysiologic background of ischemic strokes despite anticoagulation and address the question of suspected differing etiology that is not clarified yet.

## Materials and Methods

In a first step, the histologic thrombus composition of an existing collection of anterior circulation thrombi was analyzed depending on prior anticoagulation and with respect to the determined underlying stroke etiology.

In a second step, imaging thrombus characteristics of an independent cohort were compared depending on prior anticoagulation and with respect to the determined underlying stroke etiology.

The local ethics committee gave a positive vote to analyze histologic (5518/12) and imaging (250/17S) thrombus characteristics. For patients with the histologic analysis, informed consent of the patients was obtained. Need for informed patient consent for retrospective imaging analysis was waived in accordance with the ethical standards of the 1964 Declaration of Helsinki and its later amendments.

### Sample and Patient Description

The histologic cohort (*n* = 122) consisted of patients with a large vessel occlusion of the anterior circulation who were treated at a single comprehensive stroke center between October 2010 and September 2012 and whose thrombi were collected during the endovascular treatment therapy [cohort already described in Boeckh-Behrens et al. (13)].

The independent imaging cohort (*n* = 101) consisted of a homogenous collective of patients with an acute occlusion of the middle cerebral artery, treated at the same single comprehensive stroke center between January 2015 and December 2017 after applying specified exclusion criteria as described in a previous work (11).

In each cohort, patients with a determined cause of stroke were identified [Trial of ORG 10172 in Acute Stroke Treatment (TOAST) group 1, 2, and 4 (14)] and categorized as suspected cardioembolic or non-cardioembolic. TOAST group 1 (large-artery atherosclerosis) and 4 (stroke of other determined etiology) were categorized as non-cardioembolic. TOAST group 2 (cardioembolism) was categorized as cardioembolic. Patients with an undetermined cause of stroke (TOAST 5), which includes patients with more than one possible stroke etiology, were excluded from the analysis. Imaging and histologic characteristics of thrombi of suspected cardioembolic origin were analyzed in respect of prior anticoagulation and compared with non-cardioembolic thrombi without anticoagulation. Thrombi with insufficient anticoagulation were analyzed separately.

The prospectively collected clinical, imaging, and histologic data were retrospectively analyzed and partly described before (11, 13). Basic demographic, clinical, and interventional data of patients were gathered. The National Institutes of Health Stroke Scale (NIHSS) score was assessed by NIHSS-certified neurologists at the time of admission and at time of discharge. The modified thrombolysis in cerebral infarction (mTICI)–score (15) was determined by two experienced neuro-interventionalists in consensus. All patient charts were reviewed for the presence (OAC+) or absence (OAC–) of prior anticoagulation. Sufficient anticoagulation was defined as drug intake according to the recommended prescription. Insufficient anticoagulation was defined as an intake of direct anticoagulation in a lower dose than formally required by prescription rules and delayed intake but latest within 48 h or with an international normalized ratio in a therapeutic range of 1.5–1.9 for vitamin-K-antagonists.

Stroke pathogeneses were determined according to the international TOAST classification (14) based on work-up of stroke etiology which includes Holter-monitoring, laboratory analysis, an ultrasound of the brain supplying blood vessels, and a cardiac echocardiography among others (2).

### Histologic Analysis of Cerebral Thrombi

Within the histologic cohort, a quantitative analysis of the main components count was performed. Therefore, formalin-fixed and paraffin-embedded thrombi were sectioned in 2-μm-thick slides followed by the H&E staining and high-resolution scanning of the slides as previously described (16). Based on the color-guided segmentation, a quantitative analysis of the main components (fibrin/platelet, red blood cell, and white blood cell as fractions in percentage) was done using custom-made quantification software (CAMPThrombus 1.0), which has been previously explained in more detail (17). In the case of thrombus fragmentation, all fragments were included in the histologic analysis.

### Thrombus Perviousness Assessment

Within the perviousness cohort, the thrombus perviousness parameter void fraction ε was assessed for each patient in admission CT imaging as described in Berndt et al. (11). In a first step, change in thrombus attenuation Δt was measured by the increase of Hounsfield units (HU) between the native CT (native clot density) and CTA scan after automatic alignment using rigid registration method. The value of ε was calculated as the ratio of Δt within the thrombus and in the contralateral artery.

### Statistical Analysis

Quantitative histologic data of thrombi were compared between the groups with and without anticoagulation as well as between etiologic subgroups by means of nonparametric tests (Wilcoxon rank-sum tests) or Student's *t*-test, according to required test criteria. Baseline data were compared with Student's *t*-test or exact Fisher's test for categorical variables. The values of *p* less than 5% were considered as statistically significant. All statistical analyses were performed using the IBM SPSS Statistics (version 26, IBM Corp, Armonk, NY).

## Results

### Baseline Characteristics

**Figure 1:**
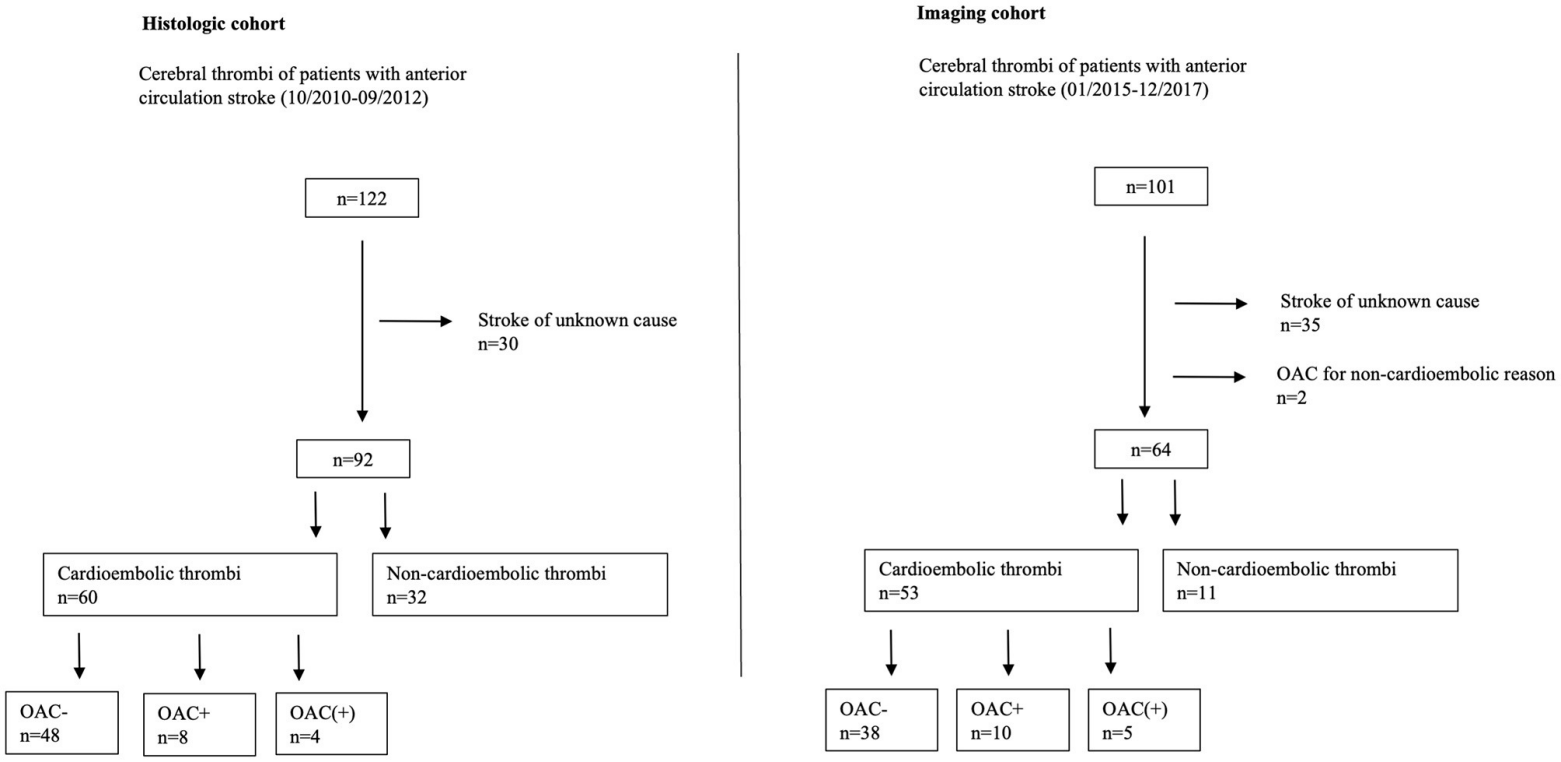
Study flowchart. OAC– no prior oral anticoagulation. OAC(+) prior insufficient oral anticoagulation. OAC+ prior oral anticoagulation. *n*-number.

In total, 92 patients were identified appropriate for the analysis of thrombus histology (histologic cohort) and 64 patients for the assessment of cerebral thrombus perviousness (imaging cohort). Patient numbers and reasons for the exclusion in both cohorts are summarized in the study flowchart presented in Figure 1. Clinical baseline characteristics of thrombi of a suspected cardioembolic origin are displayed in Table 1 for the histologic and imaging cohort, each according to the presence or absence of prior OAC. Baseline data of all thrombi without stratification for prior anticoagulation were presented before (11, 13).

**Table 1:** Baseline characteristics of patients with suspected cardioembolic stroke in respect of prior anticoagulation for the histologic and imaging cohort.

	**Histologic cohort**		***p*-value**	**Imaging cohort**		***p*-value**
	**OAC−**	**OAC+**		**OAC−**	**OAC+**	
	**(*n* = 48)**	**(*n* = 8)**		**(*n* = 38)**	**(*n* = 10)**	
Age in years (median, IQR)	78 (72.25–85)	77 (71.25–83.75)	0.9	79 (73.75–86.25)	85 (79.25–88.5)	0.1
Sex, female n (%)	35 (72.9)	2 (25)	<0.05	19 (50)	6 (60)	0.7
TICI Score >2b n (%)	40 (83.3)	7 (87.5)	0.6	32 (84.2)	9 (90)	0.6
Bridging thrombolysis n (%)	40 (83.3)	0 (0)	<0.05	15 (39.75)	0 (0)	<0.05
NIHSS at admission (median, IQR)	16 (11–18)	19 (12–24)	0.2	15 (9–17.25)	17 (13.5–20)	0.2
NIHSS at discharge (median, IQR)	8 (2–16)	6 (2–12)	0.4	4.5 (0-12)	11 (5.75-14.5)	0.2
**Type of prior anticoagulation**						
–DOAC	0 (0)	5 (62.5)	<0.05	0 (0)	9 (90)	<0.05
–VKA	0 (0)	3 (37.5)	<0.05	0 (0)	1 (10)	<0.05

*DOAC, direct oral anticoagulation; INR, international normalized ratio; IQR, interquartile range; NIHSS, National Institutes of Health Stroke Scale; n, number; OAC+, prior oral anticoagulation intake; OAC–, no prior oral anticoagulation; TICI, thrombolysis in cerebral infarction; VKA, vitamin K antagonist*.

### Histologic Thrombus Characteristics—Impact of Etiology and Prior OAC

**Figure 2:**
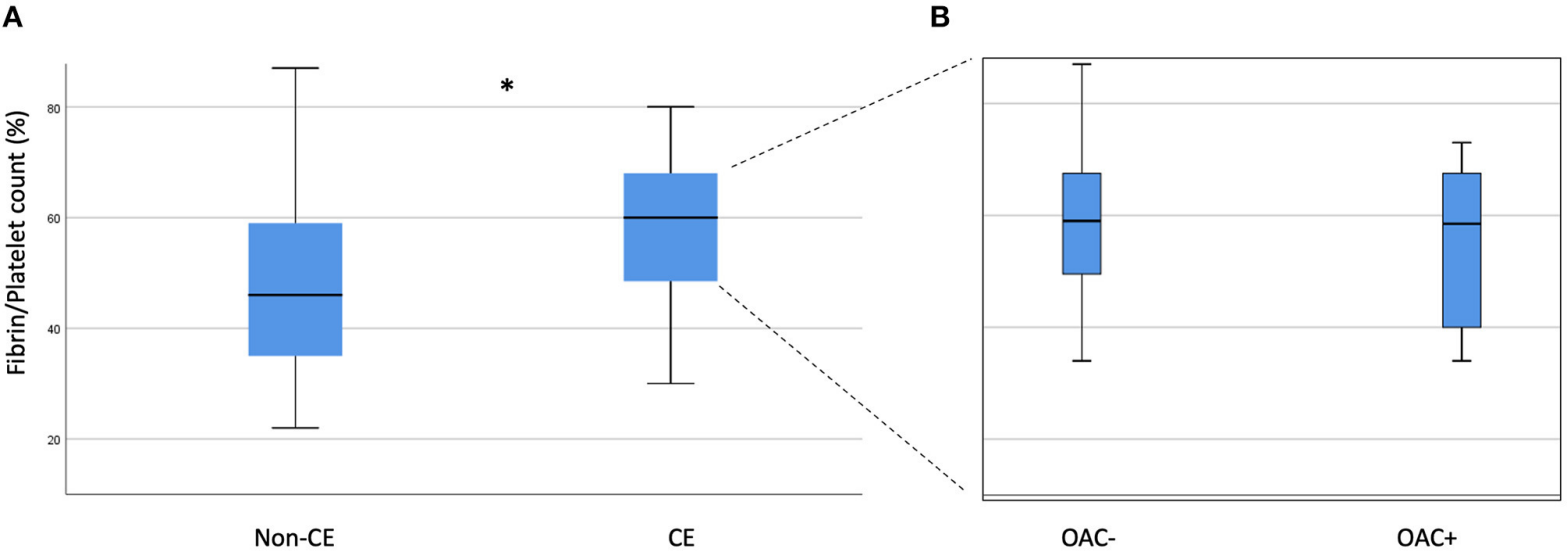
Thrombus histology of non-cardioembolic and suspected cardioembolic thrombi with focus to prior anticoagulation. **(A)** Comparison of non-cardioembolic (Non-CE) and suspected cardioembolic (CE) thrombi. **(B)** Among cardioembolic thrombi comparison of thrombi without (OAC–) and with (OAC+) prior anticoagulation. No statistical significant difference was observed. * indicates *p* < 0.05.

Compared with non-cardioembolic thrombi (*n* = 32; mean/SD 48.9% ± 17), suspected cardioembolic thrombi (*n* = 60) present with a higher fibrin/platelet content (57.2% ± 13; *p* = 0.01; Figure 2A). For the subgroup of OAC– thrombi, this difference reached a statistic level of significance (*n* = 48; 56.6% ± 13; *p* = 0.02), while in OAC+ thrombi, the higher fibrin/platelet count compared with non-cardioembolic thrombi was non-significant different (*n* = 8; 57.3% ± 13; *p* = 0.1).

Fibrin/platelet content of suspected cardioembolic OAC– thrombi (56.6% ± 13) did not differ from OAC+ thrombi (57.3% ± 13; *p* = 0.8; Figure 2B).

Red blood cell count (35.8% ± 15 vs. 37.3% ± 15; *p* = 0.9) and white blood cell count (7.4% ± 6 vs. 5.5% ± 4; *p* = 0.2) did not differ between OAC– and OAC+ thrombi of suspected cardioembolic origin.

### Imaging Characteristics of Cerebral Thrombi—Impact of Etiology and Prior OAC

**Figure 3:**
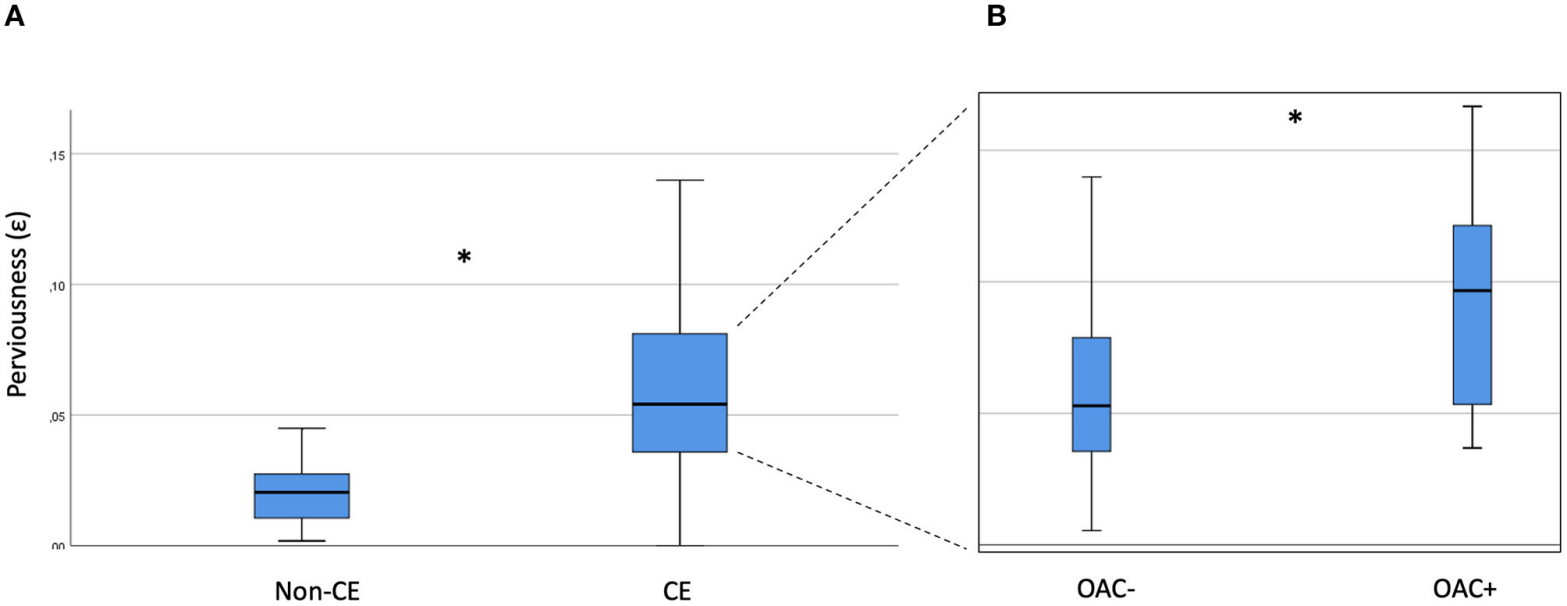
Thrombus perviousness of non-cardioembolic and suspected cardioembolic thrombi with focus to prior anticoagulation. **(A)** Comparison of non-cardioembolic (Non-CE) and suspected cardioembolic (CE) thrombi. **(B)** Among cardioembolic thrombi comparison of thrombi without (OAC–) and with (OAC+) prior anticoagulation. * indicates *p* < 0.05.

Compared with non-cardioembolic thrombi (*n* = 11; mean/SD 0.02 ± 0.02), suspected cardioembolic thrombi (*n* = 53) present with a higher thrombus perviousness (ε) (0.06 ± 0.04; *p* < 0.001; Figure 3A). Thrombus perviousness of OAC+ suspected cardioembolic thrombi (*n* = 10; 0.09 ± 0.06) is significantly higher than in non-cardioembolic thrombi (0.02 ± 0.02; *p* < 0.001).

In thrombi of suspected cardioembolic origin with anticoagulation, thrombus perviousness is significantly higher (0.09 ± 0.06) than in thrombi without anticoagulation (*n* = 38; 0.06 ± 0.03; *p* = 0.04; as shown in Figure 3B).

The other imaging characteristics (change in thrombus attenuation Δt and native clot density) were higher for OAC+ thrombi (Δt: 17.2 ±7 HU/clot density: 59.8 ± 45 HU) than for OAC– thrombi (Δt: 14.5 ±10 HU; *p* = 0.2/clot density: 43.2 ± 10 HU; *p* = 0.4), but without statistic level of significance.

### Characteristics of Thrombi With Insufficient OAC

**Figure 4:**
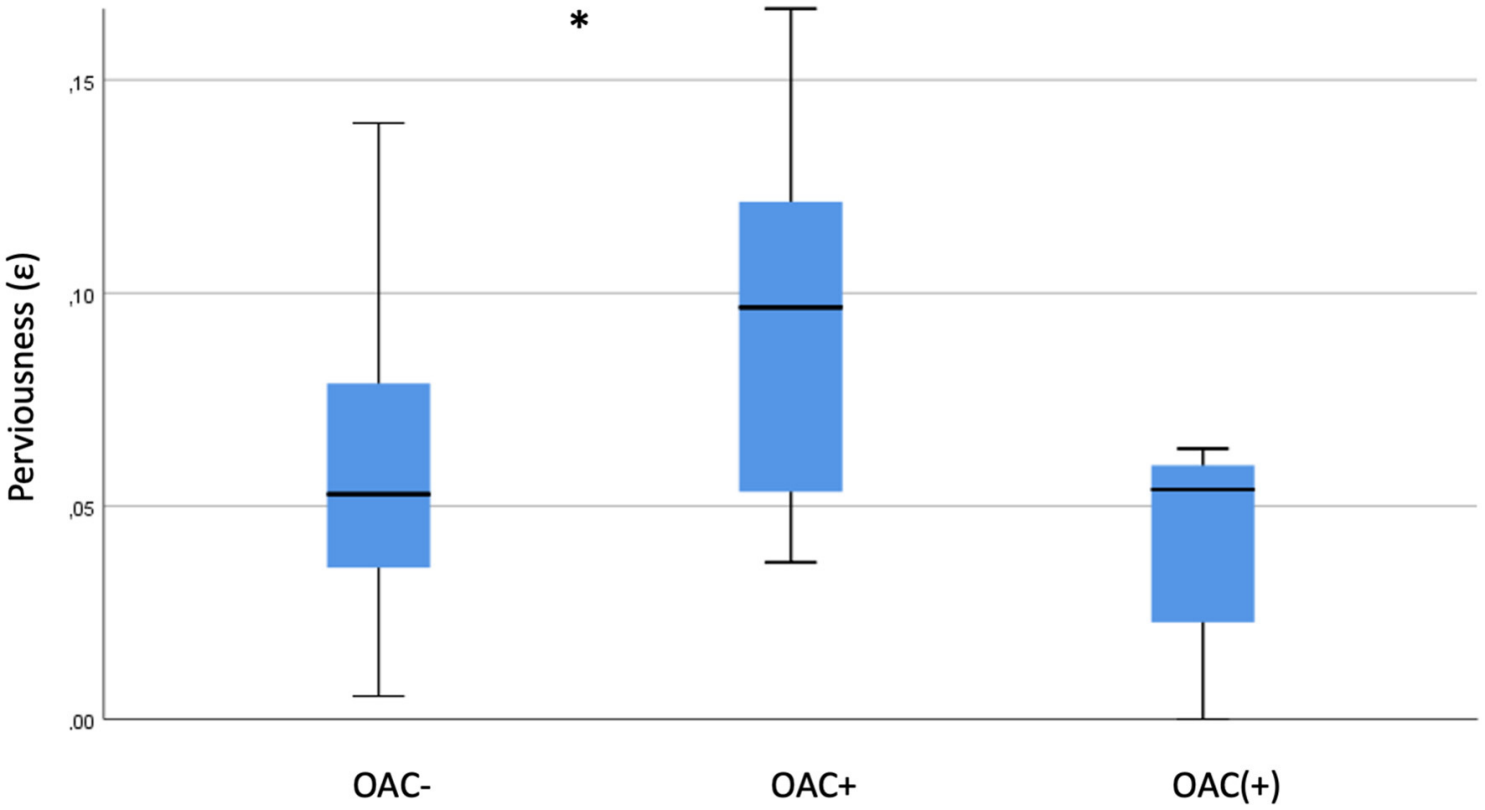
Perviousness of thrombi with prior insufficient anticoagulation. Comparison of thrombi without (OAC–), with (OAC+) and with insufficient [OAC(+)] prior anticoagulation. * indicates *p* < 0.05.

Thrombus perviousness (ε) in thrombi with insufficient anticoagulation (*n* = 5; 0.04 ± 0.02) was similar to thrombi without anticoagulation (0.06 ± 0.03; *p* = 0.4), but lower compared with thrombi with full anticoagulation (0.09 ± 0.06; *p* = 0.07; as shown in Figure 4). Fibrin/platelet content of thrombi with insufficient anticoagulation (*n* = 4; 63.5% ± 14) did not differ to other thrombi with (57.3% ± 13; *p* = 0.6) or without (56.6% ± 14; *p* = 0.3) anticoagulation.

## Discussion

The present study investigates thrombus characteristics of suspected cardioembolic thrombi with respect to prior anticoagulation. The main findings are the following:

(i) Cerebral thrombi of patients with anticoagulation for a suspected cardioembolic source had the same histologic composition as thrombi without anticoagulation, but both differ from non-cardioembolic thrombi.
(ii) Thrombus perviousness was increased in thrombi of suspected cardioembolic origin in case of prior anticoagulation. Additionally, anticoagulation might have a dose dependent effect on thrombus perviousness.

This means that the overall composition of the key clot components would not be altered by anticoagulation. At first sight, this seems to be in contrast to the clear differences regarding the perviousness as an association between histology and perviousness is known. However, the differing perviousness in case of prior anticoagulation may just reflect an alteration of the thrombi on the microstructural level regarding clot—or fibrin—tissue.

Thrombi of patients with a suspected cardioembolic stroke treated with anticoagulation were not similar to non-cardioembolic thrombi in their histologic composition. Based on this histologic analysis, the theory that cerebral ischemic strokes in patients with prior anticoagulation to prevent cardioembolism are caused by another but so far undetected embolic source is not supported. Other causes for stroke occurrence despite anticoagulation in these patients may be discussed, such as non-adherence to medication or genetic predisposition (4, 6, 7). Though appropriate secondary prophylaxis remains challenging in patients with an ischemic stroke despite prior anticoagulation, this histologic finding seems to reassure common clinical practice to continue anticoagulation without further evidence to add a second drug class, such as a platelet inhibitor, which is primary indicated in stroke of arterioembolic origin.

To our knowledge, this is the first study investigating the effect of anticoagulation on cerebral thrombus composition. One previous study reported subgroups depending on prior anticoagulation (18), with a higher fibrin count in patients with anticoagulation, however, the results are based on a smaller sample size and show a remarkable high fibrin count. The meaning of that finding was not interpreted further but reported in a subgroup only.

According to the presented data, thrombus perviousness as an imaging-based parameter of thrombus characteristics is increased in patients with prior anticoagulation. This finding is in line with *ex vivo* studies, which showed that anticoagulation alters clot permeability (19–22). In the present study, histologic analysis showed that overall clot composition is not altered, however, perviousness is increased in the case of prior anticoagulation. This could be explained by an effect of anticoagulation on the microstructure of clots, especially on the fibrin tissue. It can be suspected that a solution of fibrin bridges by anticoagulation would result in an increased perviousness of the clots.

So far, no study particularly investigated that question for cerebral thrombi of patients receiving anticoagulation based on imaging parameters *in vivo*. Only Kufner et al. reported a subgroup of patients with prior anticoagulation with no observable impact of anticoagulation on thrombus perviousness irrespective of stroke etiology (12). However, the analysis did not focus on stroke etiology and cardioembolic thrombi. This could mask the observed effect of anticoagulation on perviousness, as thrombus origin is known to have a main impact on thrombus characteristics (11, 12).

The fact that perviousness tends to be lower in patients with insufficient anticoagulation underlines the results and raises the possibility of a drug effect by the anticoagulation. In total, the impact of anticoagulation on thrombus perviousness seems to be of relevance, as (i) the determination of stroke etiology based on imaging parameters gets challenging if prior OAC intake alters clot characteristics, (ii) neurointerventional procedure may be altered by anticipated thrombus composition regarding applied devices. Interestingly, endovascular reperfusion rates are shown to be similar between patients with and without anticoagulation (23), though a recent study suggested better success rate in patients treated with direct OAC (24). A larger analysis of thrombus characteristics comparing thrombi of patients with direct OAC to patients with vitamin-K-antagonist will be of interest to clarify, if this neurointerventional observation is based on a drug specific pathophysiologic effect.

Beneath the retrospective and monocentric study design, our study has further limitations. Main limitation is the small number of patients in the cohort and its subgroups that reduces the power of the analysis and limits the validity of the presented results despite reaching the significance level. Due to the fact, the results were remarkable despite the sample size, we considered the findings should be presented to give a new impact for discussions in the stroke community and for constructing larger trials. For that reason of small groups, it was not possible to consider detailed coagulation parameters for statistical analysis, which should be evaluated in future studies. Next, the histologic and imaging groups do not have the same clinical characteristics and subgroups as these are independent cohorts, which were gathered during different timeframes. However, in both cohorts, patients were consecutively assessed to avoid bias in the study. Further, the histologic analysis was restricted to an evaluation of the relative thrombus composition. Thrombus microstructure itself—which may be altered by anticoagulation—was not investigated. Given the fact that imaging perviousness was affected by anticoagulation, such analysis would be important for a further pathophysiologic understanding. In that context, a control group of OAC+ patients with non-cardioembolic stroke (with an anticoagulation for a non-cardioembolic reason) would be helpful for future studies. Further, as thrombi of patients with posterior circulation stroke differ from anterior circulation thrombi (25), the results cannot be generalized for the posterior circulation, but further studies are required in future. Last, we investigated thrombi of patients with a large vessel occlusion, but strokes despite anticoagulation may also appear as a minor stroke without large vessel occlusion. This patient group was not studied as thrombus characteristics cannot be assessed.

## Conclusion

The present study indicates that ischemic strokes in patients with sufficient anticoagulation may not necessarily be caused by another so far undetected non-cardioembolic source. At the first sight, contradictory results of the histologic and imaging analyses might be explained by an effect of anticoagulation on the clot microstructure and not on the overall clot composition of cardioembolic thrombi. Anticoagulation seems to have an effect on thrombus perviousness, which may be of relevance for the diagnostic work-up concerning the choice of secondary prophylaxis and thrombectomy procedure in future. Given the small and exploratory approach of our study, larger studies on that topic must be performed. Though the thrombus analysis alone will not be sufficient to explain the stroke etiology alone, it may help to better understand pathophysiology of stroke despite anticoagulation and improve secondary prophylaxis to prevent stroke recurrence.

## Data Availability Statement

The raw data supporting the conclusions of this article will be made available by the authors, without undue reservation.

## Ethics Statement

The studies involving human participants were reviewed and approved by Ethikkomission, Klinikum rechts der Isar der TU München. Written informed consent for participation was not required for this study in accordance with the national legislation and the institutional requirements.

## Author Contributions

BI and MB contributed to the study concept and design. BI, TBB, CM, JH, MHP, CZ, SW, and MB participated in the data acquisition and analysis. BI, MB, and TBB wrote the manuscript with contributions from all authors.

## Conflict of Interest

The authors declare that the research was conducted in the absence of any commercial or financial relationships that could be construed as a potential conflict of interest.

## Publisher's Note

All claims expressed in this article are solely those of the authors and do not necessarily represent those of their affiliated organizations, or those of the publisher, the editors and the reviewers. Any product that may be evaluated in this article, or claim that may be made by its manufacturer, is not guaranteed or endorsed by the publisher.
